# The British Medical Association and the O'Hara Case

**Published:** 1900-09-01

**Authors:** 


### THE BRITISH MEDICAL ASSOCIATION AND THE O'HARA CASE.

The so-called "O'Hara case" appears likely to cause trouble to the British Medical Association. The affair itself was in the beginning simple enough. A Mr. O'Hara, who was a member of the British Medical Association, and of the Victorian branch thereof, was stated to be connected with a company which, in turn, was stated to be concerned in supplying certain appliances for hindering conception. Where lay the truth we do not pretend to know, but at any rate the case was brought before the Branch Council, and a motion for Mr. O'Hara's expulsion was passed. This, in due course, came for confirmation before a general meeting, which refused to confirm the action of the Council, the members of which thereupon resigned. On this a new Council was elected, and the branch was thus saved from breaking up. But considerable bitterness seems to have reigned ever since, and now by direction of the new Council, the secretary has addressed a letter to the *Australian Medical Gazette* protesting against certain *ex parte* statements made by members of the late Council. In this letter it is stated that "there were many influences at work which prevented the accused from having a fair and impartial hearing before the late Council," and that to a large number of the members the proceedings and tactics of the late Council "were abhorrent, as they must be to all right-minded men." This is pretty strong language to use in an official letter. Meanwhile the New South Wales Branch has taken the matter up, and has resolved that "a *prima facie* case for investigation has been made out, and that the Council of the British Medical Association be requested to go fully into the circumstances which led to the resignation of the late Council of the Victorian Branch." Probably the General Council will fight shy of this thorny proposition. In a previous expulsion case which occurred two or three years ago it was an easy matter to please the Australians. In that case, what with political passion and trades union feeling, the profession in Adelaide had been worked up to practical unanimity, and for the Association here to obey their behests only involved a little injustice and the kicking of a couple of men who were already down; so they expelled the blacklegs. A simple affair to a great scientific association. Now it is different. Not only are there two parties in Victoria, but the legally-elected Victorian Branch Council is on the verge of quarrelling with the New South Wales Branch Council for its impertinent interference, so that whichever side the Central Council takes it is bound to make enemies. Moreover, it is in an additional dilemma in that if it adopts the course natural to an official body, of supporting the existing and duly appointed officials in Victoria, it runs the risk of having it thrown in its teeth on every side that it is giving its countenance and support to some very objectionable practices; while, if it supports the other side, it will be charged with overriding a constitutionally elected body. Truly it is in a corner.

